# Response of species dominance and niche of plant community to wetland degradation along alpine lake riparian

**DOI:** 10.3389/fpls.2024.1352834

**Published:** 2024-03-25

**Authors:** Shengnan Wu, Shikui Dong, Ziying Wang, Shengmei Li, Chunhui Ma, Zhouyuan Li

**Affiliations:** School of Grassland Science, Beijing Forestry University, Beijing, China

**Keywords:** Qinghai Lake riparian, plant dominance, community structure, niche breadth, niche overlap, network analysis

## Abstract

Alpine wetland degradation threatens riparian biodiversity and ecological balance. Our study, conducted in July 2020 along the northern and eastern shores of Qinghai Lake, seeks to unravel the impacts of such degradation on plant species dominance and ecological niches, using advanced network analysis methods to explore the dynamics and survival strategies of plant species. We applied a space-to-time method to delineate three wetland degradation stage: a healthy swamp wetland, a slightly degraded wet meadow, and a degraded dry meadow. Six representative sampling points were chosen. At each point, three sample lines were randomly established, radiating outward from the center of the lake wetland, with each stage of degradation meticulously examined through three replicates to assess the plant communities in terms of species composition, plant height, coverage, and abundance. The results indicated: Species such as *Kobresia tibetica* and *Leymus secalinus* exhibit remarkable abundance across various stages of wetland degradation, indicating a robust tolerance to these conditions. This observation, coupled with the complexity of plant community structures in degrading wetlands, suggests that such intricacy cannot be solely attributed to the dominance of particular species. Instead, it is the result of a diverse array of species adapting to fluctuating water levels, which promotes increased species richness. Despite the prominence of species that exhibit rapid growth and reproduction, the ecological significance of less abundant species in contributing to the community’s complexity is also notable. Changes in habitat conditions due to wetland degradation facilitate both competitive and cooperative interactions among species, highlighting the dynamic nature of these ecosystems. Our analysis shows no significant linear relationship between the ecological niche overlap values and niche widths of plant species. However, the strategies employed by dominant species for competition and resource acquisition, as observed in the ecological niche overlap networks, underscore the adaptive capacity of plant communities. These insights underscore the need for tailored restoration strategies to conserve the biodiversity of alpine lake riparian ecosystems. This research not only sheds light on the resilience and adaptability of ecosystems in the Qinghai-Tibetan Plateau but also offers valuable lessons for the conservation of similar habitats worldwide. Our findings underscore the need for tailored restoration strategies to conserve the biodiversity of alpine lake riparian ecosystems. This research not only sheds light on the resilience and adaptability of ecosystems in the Qinghai-Tibetan Plateau but also offers valuable lessons for the conservation of similar habitats worldwide.

## 1 Introduction

Alpine wetlands represent a crucial biome. These ecosystems, irrespective of their size, play a vital role in biodiversity conservation and provide essential habitats for numerous species. As the highest plateau in the world, Qinghai-Tibetan Plateau boasts vast unique alpine wetlands in the size over 1 × 10^5^ km^2^, accounting for nearly 30% of the nation’s total wetland in China (Li et al., 2016). These wetlands in the forms of lake, river and marsh (Zhao et al., 2015), are primarily distributed in the Three Rivers Source Region, the eastern and southern regions of the Qiangtang Plateau, the Gannan Plateau, and the Zoigê Plateau (Bai et al., 2004). The lake wetland on the Qinghai-Tibetan Plateau is approximately 44,000 km^2^ in size, up to 33% of the total wetland area on the Qinghai-Tibetan Plateau (Zhang and Wu, 2014).

These wetlands are distinctive, endowed with irreplaceable ecological, environmental, and societal functions such as water storage, water source replenishment, and climate regulation. They play vital roles in maintaining regional sustainable development, serving as critical areas for biodiversity conservation and reactors and buffers to mitigate global climate change (Liu Z. et al., 2019). Due to its vast area and complex topography, a notable characteristic of the dynamic changes in the high-altitude wetlands of the Qinghai-Tibetan Plateau is pronounced as “east-west” disparity (Li et al., 2011). In recent decades, human activities as the primary driver together with climatic factors as a secondary driver have strongly affected the alpine wetlands on the Qinghai-Tibetan Plateau (Wang, 2007; Hou et al., 2020) resulting in the massive degradation of the alpine wetlands in this region. In the northwestern Qinghai-Tibetan Plateau, warmer and wetter climate has been associated with an overall increase in lake water levels and volume, resulting in enhanced water storage in lake and marsh wetlands (Li and Sheng, 2013). In southern Qinghai-Tibetan Plateau, despite a decreasing trend in precipitation, increased runoff derived from glacier melting has led to an overall increase in water volume, facilitating the natural recovery of many degraded or deteriorating wetlands (Zhang and Wu, 2014; Liu and Liu, 2021). Conversely, in eastern Qinghai-Tibetan Plateau, such as the Zoigê Plateau, the warmer and drier climate in the past three decades have resulted in increased evapotranspiration and declining water levels, leading to a noticeable reduction in water storage in marsh wetlands (Liu Z. et al., 2019; Zhao and Shi, 2020). In the process of wetland degradation, there is a noticeable changes of vegetation composition and structure, exhibiting a succession trend from marsh to wet meadow, to dry meadow, and eventually to desert land (Li et al., 2004).

Qinghai Lake wetland is one of the seven vital wetlands in China and was registered as Wetland of International Importance through the Ramsar Convention in 1992. This wetland is renowned as the “humidifier” of the Qinghai-Tibetan Plateau and serves as a natural bulwark for the maintenance of ecological security with rich biodiversity and the prevention of desertification in the northeastern region of the Qinghai-Tibetan Plateau (Cheng et al., 2013). Since the 1950s, the alteration of land use has exerted significantly negative impacts on the Qinghai Lake’s alpine wetland ecosystem, resulting in the decrease in vegetation productivity and shifts of community structure. In recent years, environmental issues in the Qinghai Lake region such as wetland degradation, biodiversity loss, and soil desertification have gradually become more apparent, threatening the sustainability of this precious and unique wetland ecosystem in the world. There are some studies documented the changes of plant diversity and grassland degradation in the Qinghai Lake watershed (Zhu et al., 2006; Song et al., 2011; Cheng et al., 2013). While there has been limited information regarding the changes of vegetation composition and structure with wetland degradation in the Qinghai Lake vicinity. Therefore, there is an urgent need to conduct the studies about the responses of vegetation to the degradation of Qinghai Lake wetland in the dimensions of plant species dominance and niches, the key determining indicators of community composition and structure. The results of such studies could be served as a foundation for the best conservation and restoration practices of alpine wetlands on the Qinghai-Tibetan Plateau and similar regions worldwide.

In the realm of species dominance and niches analysis, numerous studies have been performed in the world to explain the assemble and formation of vegetation in the different biome including wetland since the early stage of the 20th century. These studies have produced the ecological niche theory to explain the inter- and intra-specific relationships for structuring the plant communities. Additionally, the ecological niche theory plays a crucial role in understanding various ecological processes, such as population evolution and community succession (Peterson and Soberón, 2012; Peng and Wang, 2016; Yang et al., 2023). For instance, niche theory can clarify the allocation of resources among species, which reflects the interplay among plant populations and implies the mechanisms shaping the community composition (Nie et al., 2020). In the context of population succession, a species with a broader ecological niche tends to exhibit lower specialization, suggesting a higher degree of competitiveness. Conversely, species with narrower ecological niches display stronger specialization, which can lead to high competitiveness in their specific habitats where they are highly adapted. However, these specialized species may face challenges in resource competition when their habitats undergo significant changes, such as those caused by climate change or environmental degradation (Zhang and Xu, 2013; Wei et al., 2015). Currently, there are two different conclusions on the relationship between width of ecological and the degree of niche overlap, i.e., linear correlation (Li et al., 2014; Wang et al., 2017; Zhao et al., 2023) and non-linear correlation (Miao et al., 2015; Feng et al., 2020). The different outcomes are highly related to environmental conditions and human interventions (Zhang and Xu, 2013). Consequently, definitive outcomes for representative ecosystems in specific regions like Qinghai-Tibetan Plateau’s alpine lake wetland require a nuanced, context-specific study. In ecological studies, the concept of a niche plays a pivotal role in understanding species interactions and environmental adaptations. The fundamental niche represents the full range of environmental conditions under which a species can theoretically survive and reproduce, absent of biotic interactions like competition or predation. In contrast, the realized niche is the subset of these conditions under which a species actually exists, considering biotic and abiotic factors in its natural habitat (Hutchinson, 1957; MacArthur, 1958; Chase and Leibold, 2003). This study primarily analyzes the realized niches of species within degraded wetland ecosystems. We emphasize that the observed variations in niche width and overlap among plant species are responses to current environmental conditions rather than alterations in their fundamental niches. Such an approach allows for a nuanced understanding of species’ adaptive strategies in the face of changing ecological landscapes.

In addition, most studies on the ecological niche are primarily focused on sole analysis of ecological niche width and niche overlap values. However, very few studies have documented the niche connection and overlap in shaping the community composition and structure. Network analysis methods possess significant potential for exploring interaction, offering a multi-dimensional understanding of relationships, and uncovering potential connections (Deng et al., 2012; Meyer et al., 2020). Hence, identifying the ecological niche width and degree of niche overlap among these populations, and employing network analysis to explore niche overlap network features, enables a deeper understanding of the roles, statuses, and interactions among various species within the plant community during the process of wetland degradation.

Alpine wetlands, vital for biodiversity across sizes, are especially crucial in the Qinghai-Tibetan Plateau’s unique environments. This study focuses on the Qinghai Lake riparian area, sets out to test three hypotheses aimed at understanding how wetland degradation affects plant species’ ecological niches and community dynamics: 1) Wetland degradation can lead to alterations in the realized niches of plant species by modifying the environmental conditions and competitive dynamics within alpine lake riparian vegetation. These changes can significantly impact the composition of species, as different species adjust their ecological roles and interactions in response to the degraded conditions; 2) Wetland degradation can facilitate species competition through increasing the ecological niche overlap of plant species, leading to a more intricate plant community structure along alpine lake riparian; 3) Increased ecological niche overlap networks with wetland degradation may enhance self-regulatory capacity of plant communities through empowering dominant species’ competition for resource acquisition. By focusing on the realized niches, we explore the degradation’s impact on species competition and community structure. Our research seeks to illuminate the processes through which degradation reshapes plant communities, with the ultimate aim of guiding restoration and conservation strategies on the Qinghai-Tibetan Plateau and beyond.

## 2 Materials and methods

### 2.1 Study area

Qinghai Lake is situated in the northeastern part of the Qinghai-Tibetan Plateau, characterized by a distinct plateau continental climate. Geographically, it spans from approximately 97°53′ to 101°13′ east longitude and 36°28′ to 38°25′ north latitude. The lake’s surface lies at an elevation of 3194 meters, with an average depth of 18 meters and a maximum depth of 26.6 meters. Its longest east-west stretch measures around 106 kilometers, while the widest north-south point extends approximately 63 kilometers, giving it a somewhat elliptical shape. The lake’s shoreline stretches approximately 360 kilometers (He, 2019). The region encompasses parts of the Haiyan and Gangcha counties in the Haibei Tibetan Autonomous Prefecture, as well as the Gonghe County in the Hainan Tibetan Autonomous Prefecture. Qinghai Lake is recognized as Wetland of International Importance and a key sanctuary for rare bird and fish species, and a natural landscape conservation area on the Qinghai-Tibetan Plateau.

The study area falls within the wetland belt on the northern riparian of Qinghai Lake, part of the Qinghai Lake National Nature Reserve. The region experiences an annual average temperature range of -4.6 to 1°C, a frost-free period lasting 117 to 118 days, an annual sunshine duration of 2800 to 3330 hours, annual evaporation rates ranging from 800 to 2000 mm, and annual precipitation levels between 291 and 575 mm (Wu et al., 2011). The wetland soils predominantly consist of hydromorphic and meadow soils, with marshy and meadow soils being the primary types (Wu et al., 2011).

### 2.2 Experimental design

**Figure 1:**
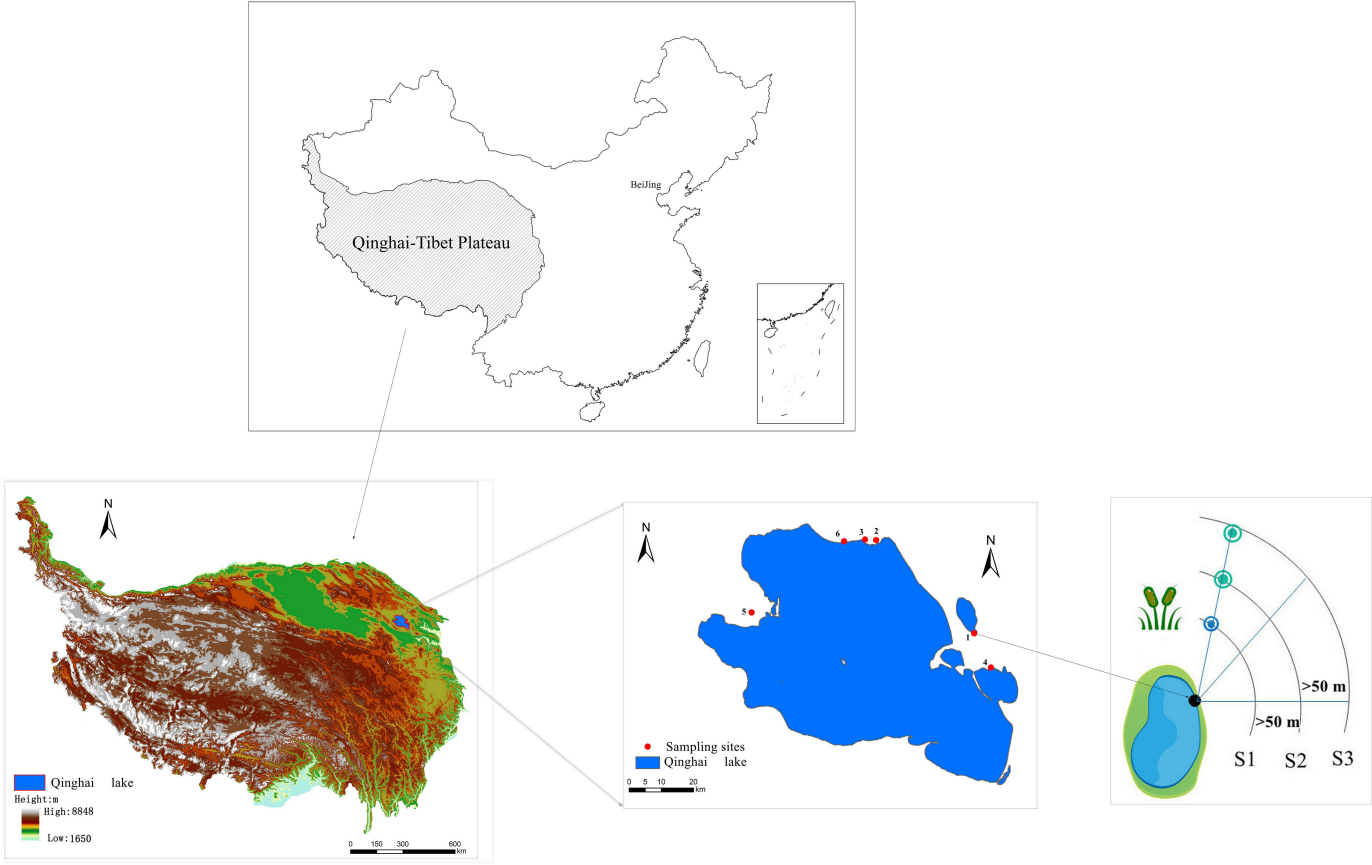
Schematic diagram of sampling point distribution and investigation in the study area.

Six representative sampling sites were selected along the northern and eastern shores of Qinghai Lake. The specific locations (**Figure 1**) include Gahai, Shaliu River, Shadao Lake, and Xiannü Bay, all of which represent typical wetland areas in the vicinity of Qinghai Lake. The common feature of these areas is that they are typical wetland ecosystems in Qinghai Lake area, which have important ecological value and research significance (Zhang and Yan, 2017). The specific geographical descriptions of the research sites can be found in **Table 1**.

**Table 1:** Geographic locations of sample points for S1stage.

Site	Elevation (m)	Longitude and latitude	
1	3197	E100°35′48.79″	N36°57′24.97″
2	3199	E100°11′48.28″	N37°12′1.82″
3	3198	E100°11′32.60″	N37°12′3.99″
4	3195	E100°39′43.42″	N36°51′53.74″
5	3200	E99°48′5.63″	N36°59′4.59″
6	3199	E100°7′5.11″	N37°11′35.71″

### 2.3 Field sampling and measurements

The degradation status of wetland vegetation was investigated following the methodology developed by Ren (Ren, 1998). By using space-to-time method, three stages of wetland degradation along Qinghai Lake riparian were defined along the lake riparian, i.e., typical marsh at the lake riparian represented the healthy wetland (S1, swamp wetland), wet meadow close to riparian represented the slightly degraded wetland (S2, swamp meadow) and dry meadow far from the riparian represented as the degraded wetland (S3, meadow). The categorization of these stages was based primarily on the characteristics of vegetation composition, with each stage typically separated by an approximate distance of 50 meters, reflecting a gradient in vegetation transition and wetland health. The sample line method was applied to sample the vegetation in the wetland riparian at different degradation stage by placing three sample lines randomly for the wetland at each stage of degradation, radiating outward from the center of the lake wetland (at the point where rivers enter the lake). Through data investigation, we have confirmed that all the points on the sampled line were swamp wetlands 40 years ago, which ensures that the spatial sampling can represent vegetation degradation. Three replicated plots of 1 m × 1 m were placed for investigating species composition, height, coverage, and the number of individuals or clumps in the plant community. To minimize potential edge effects, a minimum buffer of 5 meters was maintained between any two survey plots. The elevation and coordinates of each plot were measured using handheld GPS devices. Plant species were further categorized into functional groups of grass, sedge, legume, and forb.

Different diversity indices including Species Richness, and Importance Value (IV) were used to evaluate the structure and composition of plant communities (Agrawal and Gopal, 2012). The Importance Values for species are calculated based on the corresponding heights, cover, and frequency of each species, by averaging these relative metrics to derive the Importance Value for each species. We specifically focused on the Levins’ Ecological Niche Width Index (Bi) and Levins’ Ecological Niche Overlap Index (Oik) (Levins, 2020), which are instrumental in understanding the actual ecological space occupied by species, influenced by both biotic and abiotic factors. This distinction underscores our analysis of the realized niche, rather than the broader fundamental niche, which theoretically encompasses all environmental conditions under which a species can survive and reproduce. The ecological niche overlap values are categorized into three intervals: <0.3, 0.3-0.8, and >0.8 (Zhao et al., 2023).

### 2.4 Network analysis

In this study, a network was constructed based on the ecological niche overlap relationship matrix, and modular analysis of the ecological niche overlap network was performed. To analyze network modularity, we used the Modularity index to identify natural divisions within the network. This approach aims to maximize the density of connections within modules while minimizing connections between different modules. Various algorithms can calculate network modularity, and for our study, we identified the division that maximizes this index, thus determining the number of modules. Consistent algorithm and parameter settings were maintained throughout the study to ensure uniformity. In the analysis, nodes in the network represent plant species, with nodes in the same module tending to connect more with other nodes in the module and less with nodes outside the module (Deng et al., 2012). Species with the same color form sub-modules within the ecological niche overlap network, visually differentiated in our representations (Chen et al., 2022). The thickness of edges indicates the magnitude of ecological niche overlap values. In R, we employed the default optimal algorithm to compute the number of modules, which were then visualized in Gephi with distinct colors for each module. This visualization approach allowed us to clearly delineate and present the modular structure within our ecological network. Network modules represent species that tend to coexist in plant communities under similar habitat conditions, possibly through competition or resource sharing strategies.

### 2.5 Data processing

The calculation of the diversity index, IV, Bi, Oik and network analysis were performed using the “vegan”, “spaa”, “EcolUtils” and “igraph” packages in the program R Studio 1.1.456. The network is visualized by using Gephi 0.10 software.

## 3 Results

### 3.1 Impact of wetland degradation on plant community structure

A total of 46 species belonging to 18 families and 33 genera were collected (**Table 2**). Species with relatively high occurrence frequencies included *Leymus secalinus* and *Poa annua* from Grass functional group, *Kobresia tibetica* from sedge functional group, *Thermopsis lanceolata* from Leymus functional group. Species from the Asteraceae and Rosaceae families in the miscellaneous group also appeared frequently. The Importance Value (IV) is a comprehensive indicator of a species’ role and status within a community, reflecting its degree of dominance. There were notable differences in the primary plant composition of vegetation communities across different degradation stages (**Table 2**), indicating species turnover. In the S1 community, the dominant species were *Kobresia tibetica*, co-occurring with *Leymus secalinus*, *Carex kansuensis*, *Carex atrofusca subsp. minor*, and *Poa annua*. In the S2 stage, *Kobresia tibetica* remained the dominant species, accompanied by *Leymus secalinus*, *Elymus nutan*s, and the invasive toxic species *Thermopsis lanceolata* and the miscellaneous *Descurainia sophia*. In the S3 stage, *Leymus secalinus*, *Kobresia tibetica* are co-dominant species. Additionally, the miscellaneous species *Iris lactea* var. *chinensis* and *Potentilla anserina* were observed.

**Table 2:** Important value and niche widths of plant species in healthy wetlands (S1), slightly degraded wetlands (S2), and degraded wetlands (S3).

Species No.	Species	Importance value/Niche width (Group ID: a, b, c, d, e)		
		S1	S2	S3
		(Healthy wetland)	(Slightly degraded wetland)	(Degraded wetland)
1	*Kobresia humilis*	—	0.07/1.00 (N/A)	—
2	*Dracocephalum heterophyllum*	0.01/1.00 (b)	0.05/1.23 (b)	0.01/1.80 (b)
3	*Artemisia sacrorum*	0.01/1.80 (N/A)	—	—
4	*Agropyron cristatum*	0.19/2.51 (N/A)	—	0.05/1.00 (N/A)
5	*Descurainia sophia*	—	0.29/1.00 (N/A)	—
6	*Plantago asiatica*	0.01/1.60 (a)	0.15/1.24 (a)	0.04/1.00 (a)
7	*Elymus nutans*	0.09/2.00 (N/A)	0.48/3.09 (N/A)	0.10/5.05 (N/A)
8	*Potentilla conferta*	0.05/3.94 (a)	0.09/1.97 (a)	0.11/3.36 (a)
9	*Astragalus bhotanensis*	0.06/1.00 (b)	0.01/1.47 (b)	0.01/1.00 (b)
10	*Potentilla multifida*	—	0.04/1.00 (N/A)	0.02/1.42 (N/A)
11	*Astragalus polycladus*	—	0.08/1.00 (N/A)	0.05/2.32 (N/A)
12	*Carex kansuensis*	0.4/4.58 (c)	0.24/3.76 (c)	0.21/5.33 (c)
13	*Triglochin maritimum*	—	0.07/1.02 (N/A)	—
14	*Glaux maritima*	0.28/1.18 (N/A)	0.15/3.74 (N/A)	0.05/1.61 (N/A)
15	*Carex atrofusca subsp. minor*	0.35/2.72 (e)	0.24/2.75 (e)	0.03/1.00 (e)
16	*Gentiana pseudoaquatica*	—	0.01/100 (N/A)	—
17	*Potentilla anserina*	0.24/3.90 (a)	0.18/3.50 (a)	0.23/5.57 (a)
18	*Leymus secalinus*	0.4/2.71 (b)	0.45/4.14 (b)	0.71/5.60 (b)
19	*Oxytropis coerulea*	—	—	0.02/1.00 (N/A)
20	*Artemisia frigida*	0.04/2.48 (N/A)	—	0.13/2.27 (N/A)
21	*Oxytropis falcata*	—	0.03/1.72 (N/A)	0.03/1.00 (N/A)
22	*Gentiana squarrosa*	0.04/2.22 (a)	0.09/2.12 (a)	0.05/1.05 (a)
23	*Iris lactea* var. *chinensis*	—	0.08/1.00 (N/A)	0.51/2.95 (N/A)
24	*Silene conoidea*	0.03/1.53 (N/A)	0.03/1.00 (N/A)	—
25	*Ranunculus membranaceus*	0.06/2.90 (a)	0.08/1.95 (a)	0.11/3.12 (a)
26	*Thermopsis lanceolata*	0.26/3.76 (b)	0.37/2.22 (b)	0.13/2.95 (b)
27	*Taraxacum mongolicum*	0.13/2.22 (a)	0.09/3.07 (a)	0.15/2.97 (a)
28	*Koeleria cristata*	—	—	0.05/1.73 (N/A)
29	*Rubia cordifolia*	—	—	0.08/1.47 (N/A)
30	*Lancea tibetica*	—	0.09/1.06 (N/A)	0.01/2.37 (N/A)
31	*Mulgedium tataricum*	—	—	0.01/1.00 (N/A)
32	*Artemisia desertorum*	0.08/2.11 (b)	0.04/1.00 (b)	0.02/1.00 (b)
33	*Saussurea arenaria*	—	—	0.08/1.28 (N/A)
34	*Anemone obtusiloba subsp. ovalifolia*	—	0.03/1.53 (N/A)	—
35	*Triglochin palustre*	0.07/4.92 (d)	0.04/1.00 (d)	0.06/2.92 (d)
36	*Potentilla chinensis*	—	—	0.01/1.00 (N/A)
37	*Polygonum sibiricum*	0.09/2.74 (c)	0.01/1.00 (c)	0.02/1.18 (c)
38	*Kobresia tibetica*	0.68/4.86 (d)	0.64/6.57 (d)	0.67/5.97 (d)
39	*Primula forbesii*	—	0.03/2.51 (N/A)	0.05/1.00 (N/A)
40	*Chenopodium ficifolium*	—	0.03/1.38 (N/A)	—
41	*Kalidium foliatum*	0.03/1.00 (e)	0.03/2.80 (e)	0.01/1.00 (e)
42	*Silene galliea*	0.06/2.39 (a)	0.07/2.21 (a)	0.06/3.40 (a)
43	*Poa annua*	0.31/2.82 (N/A)	0.19/4.64 (N/A)	0.17/3.59 (N/A)
44	*Rumex crispus*	—	0.03/1.80 (N/A)	—
45	*Artemisia scoparia*	0.07/1.35 (b)	0.03/1.00 (b)	0.01/1.00 (b)
46	*Aster tataricus*	0.01/1.00 (N/A)	0.04/1.15 (N/A)	—

**Figure 2:**
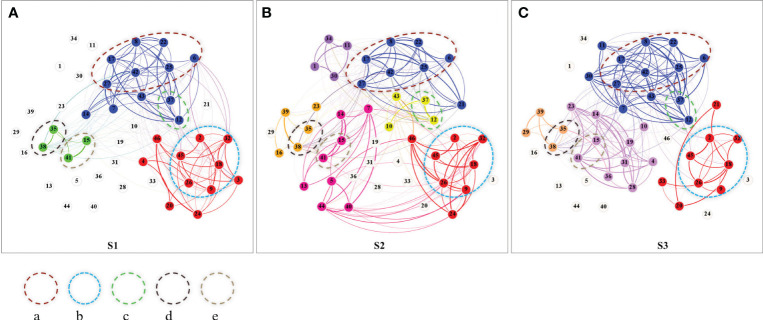
Niche overlapping networks and modules at different stages of wetland degradation (**A**: S1, (**B**: S2, **C**: S3). The numbers on the nodes are the species numbers in **Table 2**, the nodes with the same color belong to the same sub-module, and the thickness of the edge represents the niche overlap value; Dashed circles enclose groups of species with relatively stable module relationships, labeled as a, b, c, d, e based on the number of constituent species. Unconnected nodes (indicated by grey-white circles) represent species not present in the specific stage being analyzed but observed at some point during the overall survey.

The Species No. corresponds to the numerical identifiers used for plant species on the nodes in **Figure 2**, facilitating cross-referencing between the table and figure. The Group ID indicates the module group to which species are assigned based on **Figure 2**. Group ID for each stage indicate the module group to which a species belongs during that stage, as identified in the ecological network analysis. “N/A” denotes species that are present at the stage but not assigned to any of the prominent modules with relatively stable module relationships (a, b, c, d, e), and “—” indicates absence from that stage.

### 3.2 Impact of wetland degradation on species niche width and overlap

#### 3.2.1 Niche width

According to the niche metrics results, the niche width of dominant species such as Kobresia tibetica and Leymus secalinus was observed to increase in degraded wetland conditions (**Table 2**). The niche width of the dominant species *Kobresia tibetica* at the S3 stage (5.60) is greater than that at the S1 stage (4.86). The niche width of the dominant species *Leymus secalinus* at the S3 stage (5.60) is higher than that at the S1 stage (2.71). This increase, rather than indicating a shift in ecological strategy, may be more indicative of the species’ inherent tolerance to a range of habitat degradation levels. The wider niche width observed in these species at the S3 stage compared to the S1 stage underscores their adaptability to varying environmental conditions.

Some plant species exhibit non-linear relationships between ecological niche width and importance values, implying that species with low importance values may possess broader ecological niches, which could impact the growth of the dominant grasses at the same stage. For example, at the S1 stage, the perennial wetland herb *Triglochin palustre* has an importance value of only 0.07, but it possesses the largest niche width of 4.92. With the progress of wetland degradation, its niche width decreases by 79.66% at the stage S2 and 40.70% at the stage S3. At the stage S3, as one of the salt-tolerant indicators with strong preference for wet conditions, *Potentilla anserina* has an importance value of only 0.23. Nevertheless, its niche width ranks just after *Kobresia tibetica* and *Leymus secalinus*, indicating that this plant competes effectively with other plants despite it has lower importance value than other plant species at the same degradation stage of S3.

**Figure 3:**
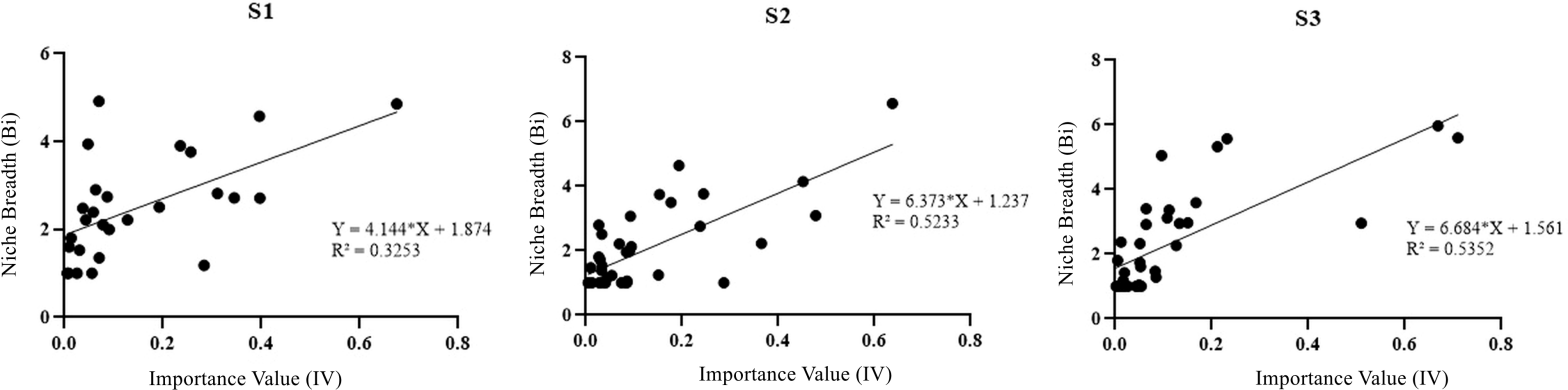
Relationship between species weight values and niche breadth species at different degradation stages.

Positive correlation between importance values and niche widths of plant species across different degradation stages was observed in regression analysis (**Figure 3**). With the increase of wetland degradation, the importance values of species become more significant in determining their resource utilization and environmental adaptation capabilities (**Figure 3**).

#### 3.2.2 Niche overlap

The overall changes in niche overlap values for the main plant species at different degradation stage are presented in **Table 3**. In general, species with niche overlap values <0.3 represent the largest proportion with decreasing trend along the wetland degradation gradient, while those with niche overlap values >0.8 represent the smallest proportion with increasing trend along the wetland degradation gradient. The ecological niche overlap index increases with wetland degradation, from 0.27 at the degradation stage of S1 to 0.43 at the degradation stage of S3.

**Table 3:** Intervals of niche overlap values for species in healthy wetlands (S1), slightly degraded wetlands (S2), and degraded wetlands (S3).

	Percentage of each interval (%)			Average niche overlap index
	Niche overlap value <0.3	Niche overlap value0.3-0.8	Niche overlap value>0.8	
S1 (Healthy wetland)	62.94%	28.82%	8.24%	0.27
S2 (Slightly degraded wetland)	61.78%	24.89%	13.33%	0.33
S3 (Degraded wetland)	47.60%	32.69%	19.71%	0.43

**Figure 4:**
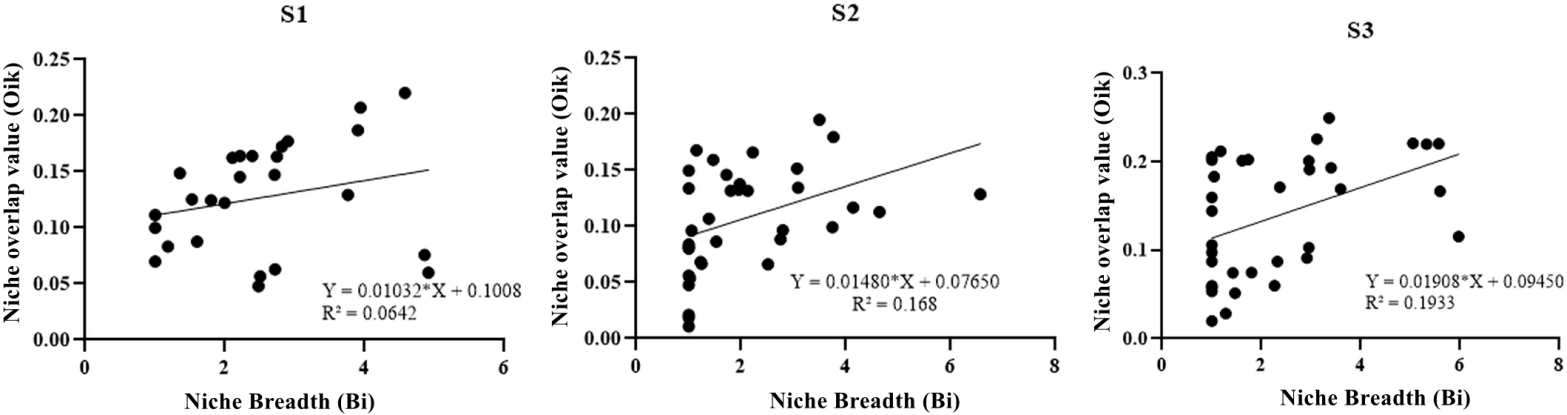
Relationship between niche breadth and niche overlap value of species at different degradation stages.

As shown in **Figure 4**, there are no significant linear relationship between the ecological niche width and niche overlap values of plant species at different degradation stages (with R^2^ values of 0.0642, 0.168, and 0.1933), implying that the species with the largest niche width may not necessarily have the highest overlap values with other plant species.

#### 3.2.3 Networks of niche overlap

As shown in **Figure 2**, the number of network modules increased from 3 at the degradation stage of S1 to 5 at the degradation stage of S3, with the highest number of modules 6 at the degradation stage of S2. There are five groups of species clusters with stable module relationships.

Group “a” consists of *Plantago asiatica* (6), *Potentilla conferta*m (8), *Potentilla anserina* (17), *Gentiana squarrosa* (22), *Ranunculus membranaceus* (25), *Taraxacum mongolicum* (27), and *Silene galliea* (42), indicating that these low-growing herbaceous plants form a stable module with shared ecological strategies. Among them, *Potentilla anserina* exhibits more developed creeping stems and higher frequency of occurrence, and a larger ecological niche width, indicating its competitive advantage in this group.

Group “b” is comprised of *Dracocephalum heterophyllum* (2), *Astragalus bhotanensis* (9), *Leymus secalinus* (18), *Thermopsis lanceolata* (26), *Artemisia desertorum* (32), and *Artemisia scoparia* (45). Among them, *Leymus secalinus*, the dominant species in the community, shows the widest ecological niche, followed by *Thermopsis lanceolata*. Other species in this group have importance values less than 0.1 and are relatively short in stature.

Group “c” consists of species *Carex kansuensis* (12) and *Polygonum sibiricum* (37). Group “d” comprises species *Triglochin palustre* (35) and *Kobresia tibetica* (38). Group “e” is composed of species *Carex atrofusca subsp. minor* (15) and *Kalidium foliatum* (41). Groups “c”, “d”, and “e” are each characterized by a sedge species and a salt-tolerant halophyte. The numbers in the above brackets are network nodes in **Figure 2**, which correspond to the “ Species No.” in **Table 2**.

## 4 Discussion

### 4.1 Responses of species dominance to wetland degradation

The changes in plant community succession are reflected in the characteristics of dominant species (Zhang and Sun, 2015). The degradation of alpine lake wetlands can result in a more complex vegetation community structure. At the severe degradation stage (S3), the dominant species shifted from sedge species *Kobresia tibetica* to the more drought-tolerant Gramineae species *Leymus secalinus* (Wang et al., 2020). The periodic flooding in moderate degradation stage leads to an increase in soil alkalinity (Liu et al., 2023), which subsequently facilitates the appearance of the salt-tolerant alkaline plant (Wang et al., 2020), i.e., *Cassia lanceolata* in this study at the S2 stage. Moreover, our findings agree with previous study that there is usually an increase in plant community diversity and a higher proportion of dicotyledonous weed species at the degradation stage of S2 (Li et al., 2012). The underlying reason for this phenomenon can likely be attributed to the transitional nature of land cover between wetland and grassland (Zhang and Sun, 2015). At the degradation stage S2, both wetland plants and grassland plants coexist, contributing to an overall increase in diversity and population. Additionally, our on-site observations suggest that the wetland vegetation at the S2 stage have experienced a substantial disturbance from grazing animals of cattle and sheep, which may improve the soil bulk density and nutrient input, promoting the availability of water and nutrients by plants and fostering the compensatory growth of wetland plants (Yang et al., 2021). Therefore, maintaining the ecotone between the marsh and meadow along the alpine lake riparian is vital to preserve biodiversity and supporting sustainable livestock management, although it is a challenge to keep the transitional phase (S2) for the wetland vegetation during their succession.

### 4.2 Responses of species ecological niche width to wetland degradation

The size of the species’ ecological niche width correlates positively with its adaptability to the environment (Chen et al., 2019; Liu X. et al., 2019). Generally, species with broader ecological niches in a community often possess higher environmental resource utilization capabilities, allowing them to more effectively adapt to specific habitat conditions. Species with wider ecological niche width values often become dominant and foundational in a given area (Wang et al., 2014). In this study, we found that an increase in habitat width for *Kobresia tibetica* and *Leymus secalinus* in the process of wetland degradation, meaning these plants are foundational and major dominant species using larger ecological niches to access essential resources.

It is generally believed that the species with larger importance values (alternatively dominance) also exhibit broader ecological niche widths (Chen et al., 2010), especially noted along the wetland degradation gradient, suggests that species with larger importance values may be better positioned to utilize available resources under these changing conditions. This correlation may reflect a response to environmental shifts rather than an inherent adaptive trait, with species importance values becoming more indicative of their current resource utilization strategies in the context of wetland degradation. However, there are some exceptional, e.g., *Osmorhiza aquatica* shows relatively smaller importance values (dominance) at the degradation stage of S1, while it demonstrates larger niche width for higher resource utilization and competitiveness. *Potentilla anserina* is characterized by lower importance values (dominance) at the degradation stage of S3, but it exhibits bigger niche width for higher resource utilization and competitiveness in the community. The presence of these species underscores that dominance is not the sole determinant of ecological niche width (Qi et al., 2011). Likely, these findings may support our first hypothesis that wetland degradation can alter the dominant species and ecological niches of plant species. These changes underscore how degradation modifies environmental conditions and competitive dynamics, significantly altering species composition within the alpine lake riparian vegetation. The adaptation of these species through expanded ecological niches reflects a dynamic response to altered habitat conditions, suggesting a complex interplay between species dominance, ecological niches, and environmental shifts, ultimately reshaping the plant community structure in degraded wetlands.

### 4.3 Responses of species ecological niche overlap to wetland degradation

Ecological niche overlap reflects the similarity and competitiveness of two or more plant populations in community environments, demonstrating their ability of resources sharing, resource utilization, and inter-species competition. Normally, ecological niche overlap arises when multiple species coexist along a resource gradient and the magnitude of overlap values indicates the similarity in resource utilization strategies among different species (Zhao et al., 2023). In this study, we observed that the average ecological niche overlap indices for all species increased consistently with degradation. These findings imply that the living spaces for meadow plants not marsh plants are improved by reduced inundation, fostering resource proximity among populations and stimulating inter-plant competition, when the alpine lake wetland are degraded. However, an excessive competition was not observed at the stages S1-S3, emphasizing that not all of species ecological niche overlap can result in competition (Feng et al., 2020). These findings support our hypothesis that observed variations in niche width and overlap among plant species are responses to current environmental conditions rather than alterations in their fundamental niches, highlighting the need for conservation efforts focused on habitat preservation and restoration in the context of wetland degradation. Our observations of shifting niche dimensions in response to environmental conditions lend support to Grinnell’s niche theory (Grinnell, 1917), while also highlighting the adaptive strategies species employ in degraded habitats.

In contrast to linear relationship between ecological niche width and overlap obtained from the studies in alpine meadow (Wang et al., 2017), semi-arid steppe (Li et al., 2014), and desert steppe (Zhang and Xu, 2013), our study revealed a non-significant linear relationship between ecological niche width and overlap across all degradation stages. This agrees with the results of studies from artificially enclosed grasslands in semi-arid regions (Liu et al., 2014; Feng et al., 2020). This suggests that in these specific environments, competitive interactions and resource utilization strategies may be more complex, requiring tailored conservation strategies to address these nuanced ecological responses. Essentially, significant ecological niche overlap values do not necessarily manifest between species with large ecological niche widths; instead, it could involve species with comparably smaller niche widths (Zhang and Xu, 2013). This divergence may stem from inherent biological differences among species, resulting in varied environmental requirements (Nie et al., 2021). Additionally, the relationship between species ecological niche width and niche overlap may be influenced by the successional stage (Zhang et al., 2016). In our study, high spatial heterogeneity in environmental resources in marsh, wet meadow and dry meadow at different degradation stage may lead to a disappearance of a pronounced correlation between ecological niche width and overlap (Zhang and Xu, 2013).

### 4.4 Responses of ecological niche overlap networks to wetland degradation

Our findings indicate a nuanced increase in network module numbers from the initial to the final stages of wetland degradation, with a peak at the S2 stage, suggesting complex species interactions and adaptation strategies. This pattern aligns with the understanding that within-module connectivity reflects shared ecological strategies and resource requirements (Deng et al., 2012; Nie et al., 2020). In present study, we observed the compensatory growth of plants facilitated by the disturbance from the grazing behavior of livestock at the degradation stage of the S2 may increase the demand for resources to intensify competition between species with similar resource utilization strategies (Liu X. et al., 2019), which is reflected in the formation of more small groups with similar resource utilization strategies in this stage. In this study, we further revealed that sedge species, specifically *Kobresia tibetica*, *Carex kansuensis*, and *Carex atrofusca*, tend to form stable subgroups with plants exhibiting distinct halophytic feature. The salt-absorbing and salt-secreting actions of these halophytes contribute to the amelioration of soil conditions (Qi and Chu, 2005), which may suggest an adaptability of the sedge functional group for alpine wetland ecosystems. In our analysis, species not consistently present in each stage or not affiliated with the same-colored module across degradation stages may represent ecological flexibility or niche specificity. Their presence or absence in certain stages or modules could reflect potential adaptation strategies to degradation conditions. However, our study primarily focuses on the dynamics and composition of relatively stable modules and their constitutive species to more clearly represent network structural dynamics. This nuanced understanding of niche dynamics offers fresh insights into species-environment interactions. Additionally, we found from this study that not all of overlap can lead to competition, which agrees with previous researchers that some species can coexist harmoniously when resources are abundant (Wang et al., 2017).

## 5 Conclusion

In conclusion, our study of Qinghai Lake’s wetland riparian degradation revealed several key insights into plant community dynamics and species responses. Firstly, species such as *Kobresia tibetica* and *Leymus secalinus* demonstrated high abundance across varying degrees of wetland degradation, indicating their strong tolerance to these environmental changes. This suggests that certain species maintain their dominance regardless of degradation levels, which is a crucial factor in understanding ecosystem resilience. Secondly, we found that the complexity of plant community structures in degrading wetlands cannot be attributed solely to the dominance of certain species. Instead, this complexity arises from the ability of a diverse range of species to thrive at different water levels, resulting in greater species richness. The presence of both dominant species, characterized by traits favoring rapid growth and reproduction, and less abundant species, each with its unique ecological niche, contributes to the intricate nature of these communities. Contrary to what might be expected, our findings indicated no significant linear relationship between the ecological niche overlap and ecological niche widths of plant species. This highlights the complexity of species interactions and niche dynamics in the context of environmental degradation. Lastly, the observed ecological niche overlap networks during the degradation process shed light on the strategies employed by dominant species for competition and resource acquisition. These strategies are pivotal in enhancing the self-regulatory capacity of plant communities, a vital aspect for ecosystem stability. These findings could be served as the scientific foundations for the best conservation and restoration practices of alpine wetlands on the Qinghai-Tibetan Plateau and similar regions across the world.

## Data availability statement

The original contributions presented in the study are included in the article/supplementary material. Further inquiries can be directed to the corresponding author.

## Author contributions

SW: Formal analysis, Visualization, Writing – original draft. SD: Funding acquisition, Project administration, Writing – review & editing. ZW: Investigation, Writing – review & editing. SL: Investigation, Writing – review & editing. CM: Investigation, Writing – review & editing. ZL: Writing – review & editing.
